# CD8^+^ T-cells target the Crimean-Congo haemorrhagic fever virus Gc protein to control the infection in wild-type mice

**DOI:** 10.1016/j.ebiom.2023.104839

**Published:** 2023-10-20

**Authors:** Deepashri Rao, Kimberly Meade-White, Shanna Leventhal, Evan Mihalakakos, Aaron Carmody, Heinz Feldmann, David W. Hawman

**Affiliations:** Laboratory of Virology, Division of Intramural Research, National Institute of Allergy and Infectious Diseases, National Institutes of Health, Rocky Mountain Laboratories, Hamilton, MT 59840, USA

**Keywords:** Crimean-Congo haemorrhagic fever, CCHFV, T-cells, Mouse model, Adaptive immunity

## Abstract

**Background:**

Crimean-Congo haemorrhagic fever (CCHF) is a serious viral hemorrhagic fever caused by the CCHF virus (CCHFV). Spread by the bites of infected ticks or handling of viremic livestock, human disease is characterized by a non-specific febrile illness that can rapidly progress to fatal hemorrhagic disease. No vaccines or antivirals are available. Case fatality rates can vary but can be higher than 30%, although sub-clinical infections are often unrecognized and unreported. Yet, while most humans infected with CCHFV will survive the infection, often with little-to-no symptoms, the host responses that control the infection are unknown.

**Methods:**

Here we investigated the role of cellular immunity in control of CCHFV infection in an immunocompetent mouse model.

**Findings:**

We found that CD8^+^ T-cells are crucial for efficient control of the acute infection and rapidly acquired CCHFV-specific antiviral effector functions such as production of antiviral cytokines and degranulating in response to CCHFV peptides. We further identified the minimal CD8^+^ T-cell epitopes in the viral Gc proteins and that infection of mice lacking IFNγ resulted in worsened disease and higher viral loads.

**Interpretation:**

Together our data suggest that CD8^+^ T-cells are important for control of acute CCHFV infection likely through production of antiviral cytokines.

**Funding:**

This work was supported by the Intramural Research Program of the NIH.

**Research in context**

**Evidence before this study**

The adaptive immune responses that are required to control acute CCHFV infection are poorly understood. Humoral immunity is often absent in fatal cases of CCHF while cellular immunity is even less understood. The effector functions required of these responses to prevent fatal disease are unclear. We have previously reported a mouse-adapted variant of CCHFV and found that infected mice deficient in adaptive immunity rapidly succumbed to the infection indicating adaptive immunity is required for survival in this model.

**Added value of this study**

Using this immunocompetent mouse model we have identified a key role for cellular immunity in controlling acute CCHFV infection. Depletion of CD8^+^ T-cells resulted in prolonged clinical disease and poor viral control. Further studies found that CD8^+^ T-cells likely control CCHFV through production of antiviral cytokines rather than cytotoxic effector functions. Mice deficient in perforin exhibited worsened disease in the absence of increased viral loads suggesting host immunopathology may also contribute to clinical disease.

**Implications of all the available evidence**

Our data suggest cellular immunity is a key host adaptive response to acute CCHFV infection. However, mice depleted of T-cells still survived the infection suggesting that additional immune responses such as humoral immunity also contribute to control of the infection.

## Introduction

Crimean-Congo haemorrhagic fever virus (CCHFV) is a tickborne virus in the *Bunyavriales* order. Crimean-Congo haemorrhagic fever (CCHF) presents initially as a non-specific febrile illness that can rapidly progress to fatal hemorrhagic disease.^1^ Currently, there are no approved antivirals nor widely available vaccines. In addition to serious disease, sub-clinical or asymptomatic infections are an underappreciated clinical outcome^2^ with some serosurveys identifying high human seroprevalence in the absence of reported cases of hemorrhagic fever.^3^, ^4^, ^5^, ^6^ Why some infections result in severe disease while others are asymptomatic is unclear but likely involves contributions of host and viral determinants of pathogenesis.

A key area of limited understanding for CCHF is how host adaptive immunity controls the infection in either naïve or vaccinated hosts. Fatal cases often have no detectable CCHFV-specific antibody^7^ while survivors may not develop neutralizing antibody responses until well after resolution of acute disease. The role of cellular immunity is even less clear with increased numbers of CD8^+^ T-cells seen in fatal cases^8^ and memory responses detected in survivors.^9^ However, the effector functions required of these responses to control CCHFV are unknown. We have previously shown in a mouse model recapitulating the convalescent phase of disease that cellular immunity and IFNγ were required to prevent death.^10^ In this model depletion of either CD4^+^ or CD8^+^ T-cells resulted in increased mortality while CD4^+^ T-cells were principally responsible for the systemic IFNγ response.^10^ However, recently we showed that vaccine-mediated protection required humoral immunity while cellular immunity was dispensable for vaccine-mediated protection^11^ suggesting that humoral immunity may also contribute to control of acute CCHFV infection.

We recently developed a mouse-adapted strain of CCHFV (MA-CCHFV) that recapitulates many features of human CCHF in adult wild-type mice.^12^ This model also displayed sex-linked differences in disease severity, with male mice developing more severe disease. In this model we found that MA-CCHFV infection of mice deficient in adaptive immunity (*Rag1*^*−/−*^) resulted in a uniformly fatal disease^12^ demonstrating that adaptive immunity is required to survive the infection. In this report we sought to investigate the function of cellular immunity in control of acute MA-CCHFV infection. CD8^+^ T-cells in MA-CCHFV infected mice were rapidly activated and acquired CCHFV specific antiviral effector function while depletion of CD8^+^ T-cells lead to worsened clinical disease and impaired control of viral replication. We further identified the minimal CD8^+^ epitopes within the CCHFV Gc that could robustly stimulate CCHFV-specific CD8^+^ T-cells. In examination of mice deficient in CD8 effector functions, infection of IFNγ deficient (*Ifng*^*−/−*^) and perforin deficient (*Prf*^*−/−*^) mice but not TNFα deficient (*Tnfa*^*−/−*^) mice resulted in worsened disease. Together our data identify the dominant CD8^+^ T-cell epitope in the CCHFV Gc protein and suggest that CD8^+^ T-cells contribute to control of acute CCHFV infection through production of antiviral cytokines.

## Methods

### Biosafety and ethics

All procedures with infectious CCHFV were conducted at biosafety level 4 (BSL4) conditions in accordance with operating procedures approved by the Rocky Mountain Laboratories institutional biosafety committee. Animal experiments were approved by the Rocky Mountain Laboratories institutional animal care and use committee (protocol #2020-68) and performed by experienced personnel under veterinary oversight. Mice were group-housed in HEPA-filtered cage systems and acclimatized to BSL4 conditions prior to start of the experiment. They were provided with nesting material and food and water ad libitum.

### Mice

Mice were between 8 and 12 weeks at time of infection. Mice were purchased and used directly from Jackson Laboratories: WT C57BL6/J (stock #000664), *Ifng*^−/−^ (#002287), *Tnfa*^*−/−*^ (#005540) and *Prf*^*−/−*^ (#002407). Mice were randomly assigned to study groups. Mice were humanely euthanized according to the following criteria: ataxia, extreme lethargy (animal is unresponsive to touch), bloody discharge from nose, mouth, rectum or urogenital area, tachypnea, dyspnea or paralysis of the limbs. Body temperature was recorded using a Unified Information Devices telemetry system and UID Mouse Matrix reader plates. Mice were implanted with telemetry transponders (UCT-2112, UID) via subcutaneous implantation and mice allowed to recover for at least one-week prior to CCHFV challenge. Data were recorded continuously with a zone interval of 250 ms, 2 cycles per series and a 1s series delay. Data reported as mean of readings collected during 6-h intervals corresponding to vivarium light–dark cycles.

### T-cell depletions

Mice were treated with 200 μg of rat IgG2b isotype (clone 1–2) or with 200 μg α-CD4 (clone GK1.5), α-CD8 (clone 2.43), or both diluted in neutral pH sterile phosphate buffered saline (PBS) and administered via 100 μL intraperitoneal (IP) injections on indicated days. Antibodies were purchased from Leinco.

### Intracellular cytokine staining

Single cell suspensions from spleens were generated by passage of tissue through a 100 micron strainer. Single cell suspensions from livers were generated by passage through a 100 micron strainer and leukocytes enriched via a percoll (Sigma) gradient. Red blood cells were lysed with ACK lysis buffer (Gibco). For intracellular cytokine staining, cells were plated at 1–3 × 10^6^ cells per well. Cells were stimulated in RPMI media (Gibco) supplemented with 10% fetal bovine serum (Gibco), penicillin, streptomycin, brefeldin A (Biolegend), monensin (Biolegend) and PE-conjugated CD107a (0.5 μg/mL, Biolegend). Cells were stimulated with DMSO vehicle (Hybrimax grade, Sigma), CCHFV peptides at 1 μg/mL each peptide or PMA/ionomycin (Biolegend) for 6 h at 37 °C. Cells were then processed for flow cytometry.

### Flow cytometry

Single cell suspensions generated as above or stimulated cells were stained with Zombie Aqua viability dye (Biolegend), Fc receptors blocked with TruStain FcX (Biolegend) and then stained with fluorophore conjugated antibodies against CD45 (Clone 30-F11, BD AB_2872789), CD3 (145-2C11, BD AB_2738278), CD4 (RM4.4, Biolegend AB_2563110), CD8a (53–6.7, Biolegend AB_493423 or AB_2562610), CD44 (IM7, Biolegend AB_493679), CD69 (H1.2F3, BD AB_2740186). For intracellular staining, cells were fixed and permeabilized with FOXP3 fix/perm kit (eBioscience) and then stained with antibodies against IFNγ (XMG1.2, Biolegend AB_315404), TNFα (MP6-XT22, Biolegend AB_2629800), Perforin (S16009A, Biolegend AB_2721463), IL-2 (JES6-5H4, Biolegend AB_2650897) or Ki67 (16A8, Biolegend AB_2564285). For all presented data cells were gated by forward and side-scatter to exclude debris, doublets and non-viable cells. For all studies, T-cells were defined as CD45^+^CD3^+^, CD8 T-cells were defined as CD45^+^CD3^+^CD4^−^CD8^+^ and CD4 T-cells were defined as CD45^+^CD3^+^CD4^+^CD8^−^. Gating strategy is provided in Supplemental Figure S1. Gating for activation markers and cytokines was set using cells which were stained with antibodies except those against activation markers or cytokines or stimulated in the absence of CD107a.

### Virus stocks

MA-CCHFV used in this study is same stock as previously described.^12^ Mice were infected via the IP route.

### qRT-PCR

Viral loads were quantified by qRT-PCR as previously described.^12^ Data are shown with limit of quantification (LoQ) and limit of detection (LoD). LoQ was defined as the last standard to amplify (100 copies) while LoD was defined by extrapolating the standard curve to the copy number given by a Ct value of 40. Samples that had no amplification were set at the LoD.

### IFNγ ELISpot

IFNγ ELISpots were performed on single cell suspensions of splenocytes isolated as above using mouse IFNγ ELISpot kits (Cellular Technologies Limited, CTL). Single-cell suspensions of splenocytes were resuspended in CTL Test medium (CTL) and plated at 100,000 cells per well. 15-mer peptides overlapping by 11 amino acids spanning the entire NP and GPC (Genscript) were pooled and cells stimulated with pooled peptides at 1 μg/mL each peptide for evaluation of pools. For ELISpots stimulating cells with individual peptides, cells from indicated groups were pooled, plated at 300,000 cells per well and stimulated with each peptide at 5 μg/mL in duplicate. Cells were incubated for 24 h and then plates developed according to manufacturer protocol. T-cell truncation libraries against epitopes of interest were designed and synthesized at >90% purity and used to stimulate cells as above.

### Statistics

Statistics were calculated in Prism v9 (GraphPad) using recommended statistical tests. Sample size was based on previous experience with the MA-CCHFV model and mice randomly assigned to study groups. Researchers were not blinded to study groups and no mice were excluded from analysis.

### Role of funders

Funders had no input on study design, data collection, data analyses, interpretation or writing of report.

## Results

### Mice depleted of CD8^+^ T-cells have prolonged clinical disease following MA-CCHFV infection

**Fig. 1:**
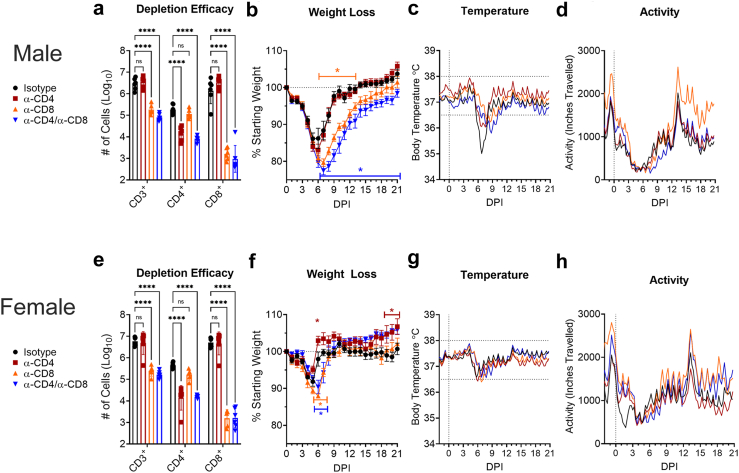
**Depletion of CD8 T-cells exacerbates clinical disease in MA-CCHFV infected mice.** Male (a–d) or female (e–h) WT mice were infected with MA-CCHFV. On days −2, +3, +10, +17 PI mice were treated with isotype or antibodies to deplete CD4, CD8 or both CD4 and CD8 T-cells. On day +6 PI a subset of mice (n = 6 per group) were analyzed by flow cytometry to evaluate depletion efficacy on total CD3+ T-cells, CD4 T-cells or CD8 T-cells (a & e). Mice were weighed daily, n = 8 per group, (b & f) and body temperature (c & g) and activity (d & h) were measured by the cage telemetry system (n = 8 per group for female α-CD4/α-CD8, n = 4 for all other groups). Statistics were performed using a two-way ANOVA with Dunnett's multiple comparisons test (a & e), or Tukey's multiple comparisons test (b & f). Data shown as mean plus SD (a & e) or mean plus SEM (b & f). ns P > 0.05, ∗P < 0.05, ∗∗∗∗P < 0.001. For telemetry data (c, d, g, h) data points were smoothed using 4 neighbors to each side and a 2nd order polynomial. Cages were changed on days −1 and 14 relative to infection.

We first evaluated the role of T-cells in control of MA-CCHFV infection by investigating the consequence of depleting either CD4^+^, CD8^+^ or both CD4^+^ and CD8^+^ T-cells on clinical disease progression. Mice were treated with antibodies to deplete the targeted cell types on days −2, 3, 10 and 17 post-infection (PI). In a subset of mice, we confirmed depletion efficacy through flow cytometry on the liver at day 6 PI and achieved significant and specific depletion of targeted T-cells in the liver (Fig. 1a and e). Depletion efficacies of targeted cell types were 91–99.9% in male mice and 95–99.9% in female mice. Compared to isotype-treated mice, male mice depleted of CD8^+^ T-cells had significantly increased weight loss from day 6 through 13 while mice depleted of both CD4^+^ and CD8^+^ T-cells had significantly increased weight loss from day 6 until at least day 21 (Fig. 1b). We also observed a trend towards increased weight loss in male mice depleted of both CD4^+^ and CD8^+^ T-cells compared to mice depleted of CD8^+^ T-cells alone (Fig. 1b) suggesting that CD4^+^ T-cells may further contribute to control of disease. However, the effect was modest. Weight loss in infected isotype-treated male mice was associated with hypothermia around day 6 and 7 PI (Fig. 1c) and depletion of T-cells appeared to moderate this effect (Fig. 1c). Male mice depleted of both CD4^+^ and CD8^+^ T-cells continued to exhibit decreased body temperature for several days after isotype treated mice returned to baseline around 37 °C (Fig. 1c). Infection of male mice also resulted in lethargy as quantified by continuous activity tracking (Fig. 1d). Depletion of both CD4^+^ and CD8^+^ T-cells resulted in prolonged lethargy compared to isotype or CD8-depleted mice further suggesting CD4^+^ T-cells may contribute to control of the infection (Fig. 1d).

In MA-CCHFV infected female mice depleted of CD4^+^ T-cells, we interestingly observed a general trend towards improved disease as evidenced by improved weight compared to isotype-treated mice (Fig. 1f) although this was only statistically significant on days 6 and 19–21 PI. Similar to male mice, depletion of CD8^+^ T-cells or both CD4^+^ and CD8^+^ T-cells led to worsened disease with increased and prolonged weight loss compared to isotype treated mice on days 6 and 7 PI (Fig. 1f). However, we did not observe a similar trend of increased weight loss in female mice depleted of both CD4^+^ and CD8^+^ T-cells compared to female mice depleted of just CD8^+^ T-cells as was measured in male mice (Fig. 1b). Even in female mice depleted of CD4^+^ and CD8^+^ T-cells, disease was milder than isotype-treated male mice (Fig. 1b and f) suggesting that even in mice lacking cellular immunity, female mice retain better control of the infection than male mice. We observed similar patterns of perturbations to body temperature and lethargy in female mice although overall these changes were milder than seen in male mice (Fig. 1g and h). Statistical analysis of telemetry data compared to respective baseline values on day 0 are provided in Supplemental Table S1. Survival in male or female mice was not significantly impacted by depletion of any T-cell subset (data not shown). Together these data suggest that CD8^+^ T-cells are the principal effector population of cellular immunity in recovery from MA-CCHFV infection.

### Mice depleted of CD8^+^ T-cells have impaired control of MA-CCHFV

**Fig. 2:**
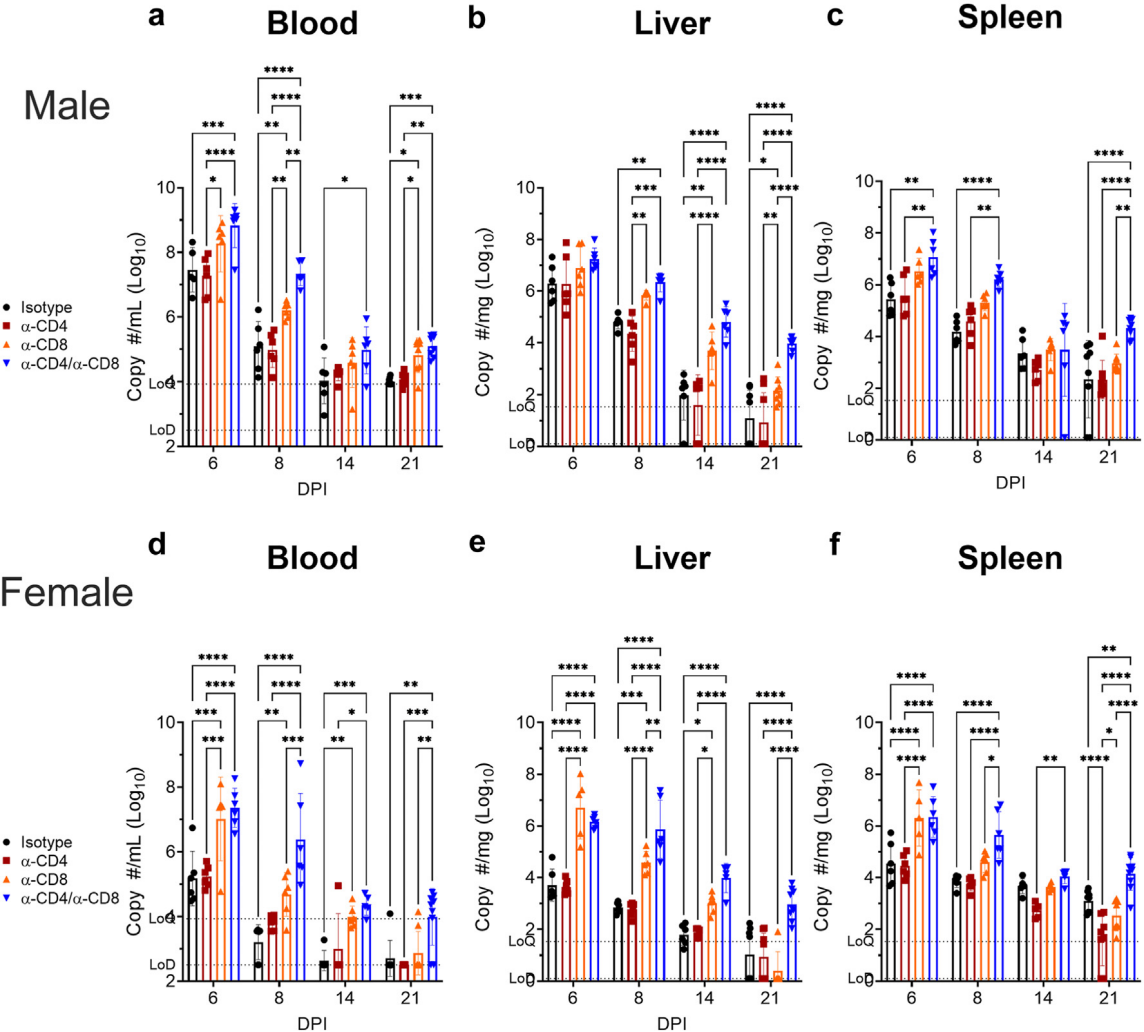
**Depletion of CD8 T-cells worsens viral control.** MA-CCHFV infected male (a–c) or female (d–f) mice treated with antibodies to deplete CD4, CD8, both CD4 and CD8 T-cells or isotype control antibody were euthanized at indicated DPI and viral loads in the indicated tissues evaluated by qRT-PCR. Data presented as mean plus standard deviation. N = 6–9 per group. Statistics calculated with two-way ANOVA with Tukey's multiple comparisons test. ∗P < 0.05, ∗∗P < 0.01, ∗∗∗P < 0.001, ∗∗∗∗P < 0.0001.

To further investigate the role of T-cells in control of MA-CCHFV, we performed a time course and collected the blood, liver and spleen for qRT-PCR to measure viral burdens at day 6, 8, 14 and 21 PI. Consistent with our data showing that depletion of just CD4^+^ T-cells largely did not impact clinical disease severity in male or female mice, compared to isotype-treated mice, mice depleted of just CD4^+^ T-cells did not have significantly increased viral RNA burdens in tissues analyzed at any timepoint (Fig. 2). Consistent with prolonged disease in mice depleted of CD8^+^ T-cells, male mice deficient in CD8^+^ T-cells had significantly increased viral RNA burdens at days 8 and 21 PI in the blood, days 8 and 14 in the liver and days 6 and 8 in the spleen (Fig. 2a–c). Male mice deficient in both CD4^+^ and CD8^+^ T-cells had further worsened viral control. Compared to isotype-treated mice, mice deficient in both CD4^+^ and CD8^+^ T-cells had significantly increased viral loads at all timepoints in the blood and liver and at days 6, 8 and 21 in the spleen (Fig. 2a–c). Further, compared to male mice depleted of just CD8^+^ T-cells, mice deficient in both CD4^+^ and CD8^+^ T-cells had significantly greater viral loads in the blood at day 8, liver at day 14 and spleen at day 21 (Fig. 2a–c). Female mice deficient in CD8^+^ or both CD4^+^ and CD8^+^ T-cells had significantly increased viral loads in the blood at days 6 and 8 PI but thereafter T-cell depleted mice had similar viral loads to isotype-treated female mice (Fig. 2d). In the liver, female mice depleted of CD8^+^ T-cells had impaired control of MA-CCHFV with significantly increased viral loads until day 14 PI, while mice depleted of both CD4^+^ and CD8^+^ T-cells had significantly increased viral loads until day 21 (Fig. 2e). In the spleen, female mice depleted of CD8^+^ and both CD4^+^ and CD8^+^ T-cells had increased viral loads at day 6 while mice depleted of both CD4^+^ and CD8^+^ T-cells had increased viral loads at day 6, 8 and 21 PI (Fig. 2f). Interestingly, depletion of just CD4^+^ T-cells lead to improved viral clearance in the spleen at day 14 and 21 PI (Fig. 2f) suggesting that CD4^+^ T-cells may impair viral clearance at later timepoints in female mice. However, compared to female mice depleted of just CD8^+^ T-cells, female mice depleted of both CD4^+^ and CD8^+^ T-cells had significantly greater viral loads in the blood at day 8, liver at day 8 and 14 and spleen at days 8 and 21 PI (Fig. 2d–f) suggesting that in the presence of an intact CD8^+^ response, CD4^+^ T-cells may also contribute to viral control. Together these data are consistent with the observed prolonged clinical disease in mice depleted of CD8^+^ or both CD4^+^ and CD8^+^ T-cells and show that CD8^+^ T-cells are required for effective control of MA-CCHFV infection.

### T-cells are rapidly activated in the liver following MA-CCHFV infection

**Fig. 3:**
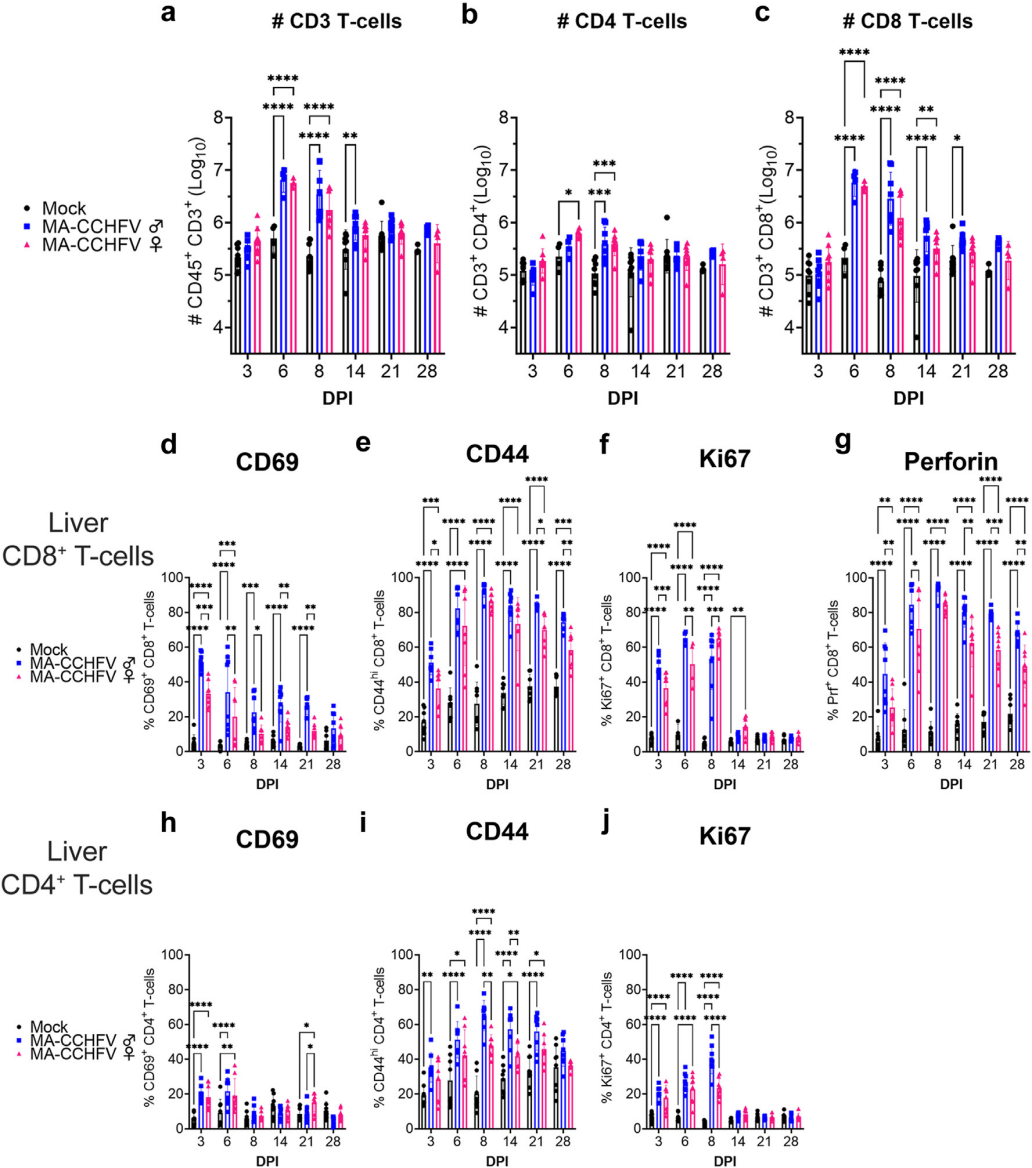
**T-cells are rapidly activated in the livers of infected mice.** Male (blue squares) or female (pink triangles) WT mice were infected with MA-CCHFV. As comparison, male and female mice also received a mock infection (black circles). At indicated time points mice were euthanized and lymphocytes isolated from the liver analyzed by flow cytometry. Total numbers of CD3^+^ T-cells (a), CD3^+^CD4^+^ T-cells (b) or CD3^+^CD8^+^ T-cells (c) in the liver were enumerated. Expression of activation markers CD69, CD44, Ki67, or perforin on CD8^+^ T-cells (d–g) or CD4^+^ T-cells (h–j) was measured and expressed as percentage of parent population positive (CD69, Ki67, Perforin) or expressing high levels (CD44) of the marker. Data presented as mean plus standard deviation. N = 4–8 per group. Statistics were calculated with a two-way ANOVA with Tukey's multiple comparisons test. ∗P < 0.05, ∗∗P < 0.01, ∗∗∗P < 0.001, ∗∗∗∗P < 0.0001.

Our *in vivo* depletion data demonstrated that CD8^+^ T-cells were the primary effector of cellular immunity in control of the MA-CCHFV infection. We next analyzed the T-cell response in the liver of MA-CCHFV infected mice by flow cytometry as the liver is a key target tissue of MA-CCHFV, showing high viral loads and substantial pathology.^12^ The gating strategy is shown in Supplemental Figure S1. We determined total number of CD3^+^ T-cells, CD3^+^CD4^+^ and CD3^+^CD8^+^ T-cells and found that by day 6 PI, MA-CCHFV infected mice had significantly increased total number of CD3^+^ T-cells that was mainly driven by significant increases in numbers of CD3^+^CD8^+^ T-cells (Fig. 3a–c). In both male and female mice infected with MA-CCHFV, the number of CD8^+^ T-cells increased by over 25-fold (Fig. 3c). Compared to mock-infected mice, increased numbers of CD8^+^ T-cells were measured in the liver until at least day 21 in male mice and at least day 14 in female mice. We also observed a slight but significant increase in CD4^+^ T-cells by day 6 in female mice and at day 8 both male and female mice had significantly greater numbers of CD4^+^ T-cells compared to mock-infected animals (Fig. 3b). The robust increase in CD8^+^ T-cells and more modest increase in CD4^+^ T-cells in the liver is consistent with our depletion data showing a greater role of CD8^+^ T-cells in control of the infection.

We next evaluated the activation status of CD8^+^ T-cells in the liver and found that CD8^+^ T-cells were rapidly activated following MA-CCHFV infection. By day 3 PI, both male and female mice had significantly increased numbers of CD8^+^ T-cells positive for the early activation marker CD69 (Fig. 3d). Compared to mock infected mice, female mice had significantly increased CD69^+^ CD8^+^ T-cells until day 6 (Fig. 3d). Interestingly, male mice had significantly increased CD69^+^ CD8^+^ T-cells until at least day 21 (Fig. 3d) suggesting ongoing T-cell activation in the livers of these mice. We also evaluated CD44 expression as a marker of antigen experienced effector cells^13^ and compared to mock-infected mice both male and female mice had significantly increased percentages of CD44^hi^ CD8^+^ T-cells in the liver as early as day 3 PI and sustained until at least day 28 PI (Fig. 3e). We evaluated Ki67 expression as a marker of cellular proliferation and both male and female infected mice had significantly increased percentages of Ki67^+^ CD8^+^ T-cells by day 3 PI and sustained to day 8 PI in male mice and at least day 14 in female mice (Fig. 3f). Lastly, CD8^+^ T-cells can exert cytotoxic function through perforin- and granzyme-mediated cell lysis^14^ and we thus evaluated perforin expression in CD8^+^ T-cells from MA-CCHFV infected mice. As early as day 3 PI, both male and female mice infected with MA-CCHFV had significant increases of perforin^+^ CD8^+^ T-cells that peaked at day 8 PI and were sustained until at least day 28 (Fig. 3g).

Although our data suggested that CD8^+^ T-cells were the primary effector cell population for control of CCHFV, we also evaluated the activation state of CD4^+^ T-cells in the liver. At day 3 and 6 PI, both male and female mice had significantly increased CD69^+^ CD4 T-cells suggesting CD4^+^ T-cells were also rapidly activated in the liver (Fig. 3h). By day 3 PI, male mice had significantly increased CD44^hi^ CD4^+^ T-cells which was sustained until at least day 21 PI. Similarly, female mice had significantly increased CD44^hi^ CD4^+^ T-cells by day 6 and sustained until at least day 21 PI (Fig. 3i). CD4^+^ T-cells in both male and female mice showed evidence of proliferation as measured by increases in Ki67^+^ CD4 T-cells by day 3 PI through day 8 PI. Overall, compared to activation of CD8^+^ T-cells in the liver, activation of CD4^+^ T-cells was more modest but followed similar kinetics.

To explore the hypothesis that worse disease in male mice is due to impaired cellular immunity, we compared T-cell activation between male and female mice. Overall, by multiple parameters infected male mice had greater magnitude of responses than female mice. Male mice exhibited greater percentages of CD44^hi^ CD8^+^ T-cells at day 3, 21 and 28 PI and greater percentages of perforin^+^ CD8^+^ T-cells at days 3, 6, 14, 21 and 28 PI compared to female mice. These data suggest that the increased disease severity in male mice is not due to impaired CD8^+^ T-cell responses.

### Humoral immunity to CCHFV is T-cell dependent

While depletion of CD4 T-cells alone did not significantly impact the progression of MA-CCHFV infection nor did it have an impact on viral loads in male mice, in male mice depletion of both CD4^+^ and CD8^+^ T-cells further worsened disease compared to just CD8 depletion alone. We hypothesized that CD4^+^ T-cells may contribute to control of the infection through support of CCHFV-specific antibody responses. Indeed, we found that mice depleted of CD4^+^ T-cells had significantly diminished CCHFV-specific IgG at day 21 PI (Supplemental Figure S2) indicating that the anti-CCHFV antibody response measured in these mice is largely CD4^+^ T-cell dependent.

### Cellular immunity elicited by MA-CCHFV infection is narrowly focused on the CCHFV NSm and Gc

**Fig. 4:**
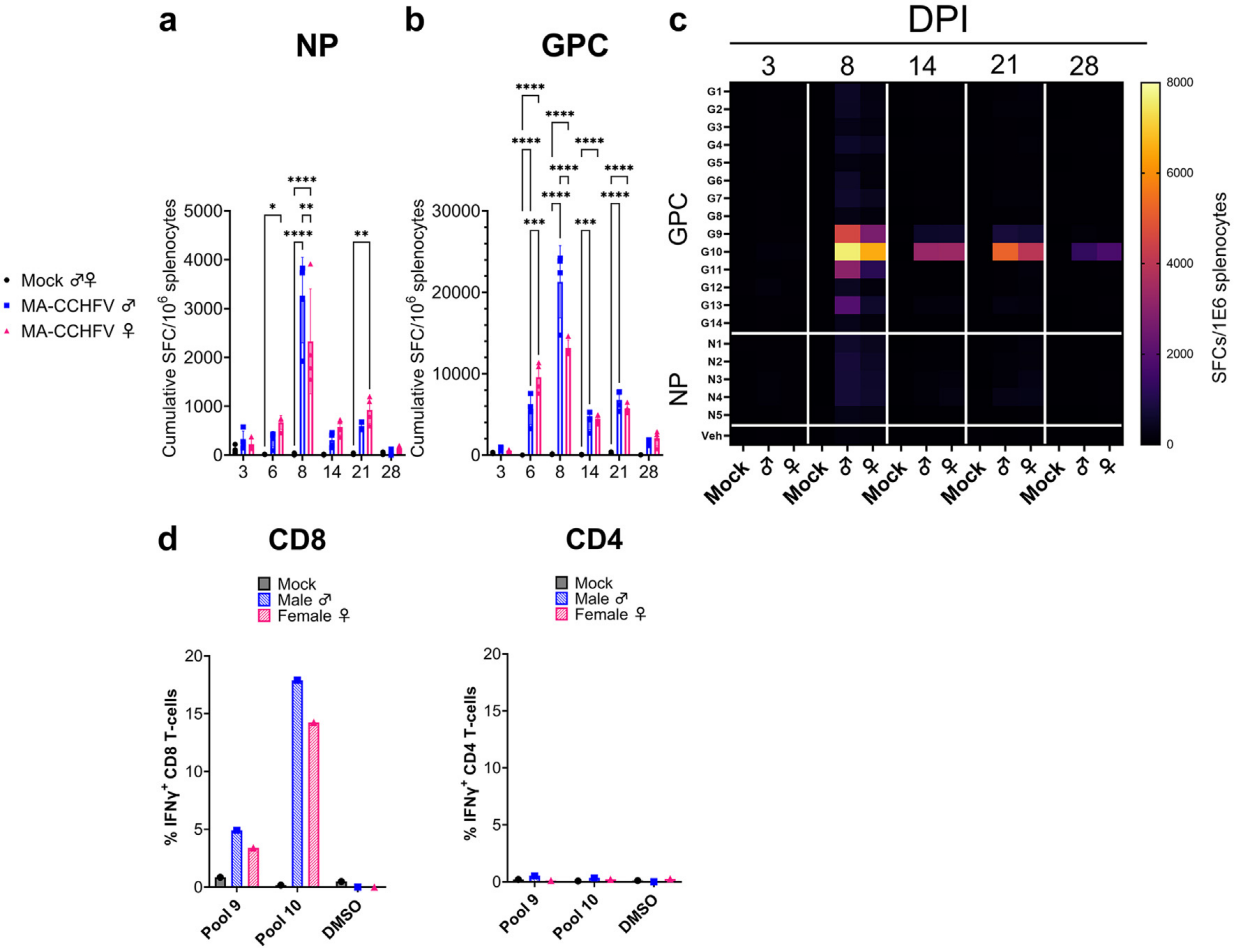
**CCHFV-specific T-cells are directed against the viral NSm and Gc.** (a & b) Splenocytes from mock- or CCHFV-infected WT mice were collected at indicated DPI and CCHFV-specific T-cell responses measured by IFNγ ELISpot. Data presented as cumulative spot forming cells (SFCs) normalized to 10^6^ splenocytes against peptide pools spanning the NP (a) or GPC (b). N = 4 per group. Data shown as mean plus standard deviation. Statistics calculated with two-way ANOVA with Tukey's multiple comparisons test. This data is shown again as the averaged (mean) response against the individual peptide pools (c). (d) Pooled splenocytes from four mock or CCHFV-infected male or female mice at day 8 were stimulated *ex vivo* with the indicated GPC pools or DMSO vehicle and percentage of IFNγ+ parent CD8^+^ or CD4^+^ T-cell populations measured by ICS. Data shown as the average of duplicate measurements of pooled cells. ∗P < 0.05, ∗∗P < 0.01, ∗∗∗P < 0.001, ∗∗∗∗P < 0.0001.

Our data showed that CD8^+^ T-cells are required for efficient control of MA-CCHFV infection and that CD8^+^ T-cells are rapidly activated in response to the infection. To investigate the kinetics of the CCHFV-specific T-cell responses we performed IFNγ ELISpot assays on splenocytes collected at days 3, 6, 8, 14, 21 and 28 PI. Splenocytes were stimulated with overlapping peptide (15-mers) pools spanning either the NP (19–25 peptides/pool) or GPC (30–31 peptides/pool). Compared to mock-infected mice, female mice had specific responses against the CCHFV NP at day 6 and 21 PI (Fig. 4a). Male mice developed significant responses against the NP by day 8 PI although these rapidly declined (Fig. 4a). Interestingly the response to peptides of the GPC were more robust. Compared, to mock-infected mice, significant CCHFV-specific IFNγ responses against the CCHFV GPC were detected as early as day 6 PI in both male and female mice (Fig. 4b). Responses peaked at day 8 PI and were maintained until at least 21 DPI. At day 6 PI, infected female mice had significantly greater responses to the CCHFV GPC than infected male mice but at day 8 PI, infected male mice had significantly greater responses against the CCHFV GPC. These data suggest male mice developed a robust T-cell response against CCHFV albeit with slightly delayed kinetics compared to female mice (Fig. 4b). When evaluating responses to specific peptide pools, responses against individual peptide pools in the GPC were stronger than NP (Fig. 4c) indicating that the majority of the CCHFV-specific T-cell response was directed against the GPC. The IFNγ response was primarily directed against peptide pools 9 and 10 of the CCHFV GPC (Fig. 4c). These pools are within the NSm (pool 9) and N-terminus of the Gc protein (pool 10). Responses against pool 10 were detectable to at least day 28 while those against pool 9 declined through day 21 and were minimal at day 28 (Fig. 4c). These data suggest memory responses are largely focused against pool 10 in MA-CCHFV infected mice. We confirmed our ELISpot findings using intracellular cytokine staining (ICS) with pooled splenocytes collected at day 8 PI and stimulated with pools 9 or 10 *ex vivo*. Our ICS data showed that pool 9 and 10 stimulated CD8^+^ T-cells to produce IFNγ (Fig. 4d) and that pool 10 resulted in a greater percentage of CD8^+^ T-cells producing IFNγ, consistent with our ELISpot data. Further, our data showed that these pools did not stimulate CD4^+^ T-cells to produce IFNγ (Fig. 4d) suggesting that the IFNγ responses measured in our ELISpot were generated by the CD8^+^ T-cells.

### Liver CD8 T-cells produce IFNγ, TNFα and degranulate in a CCHFV-specific manner

**Fig. 5:**
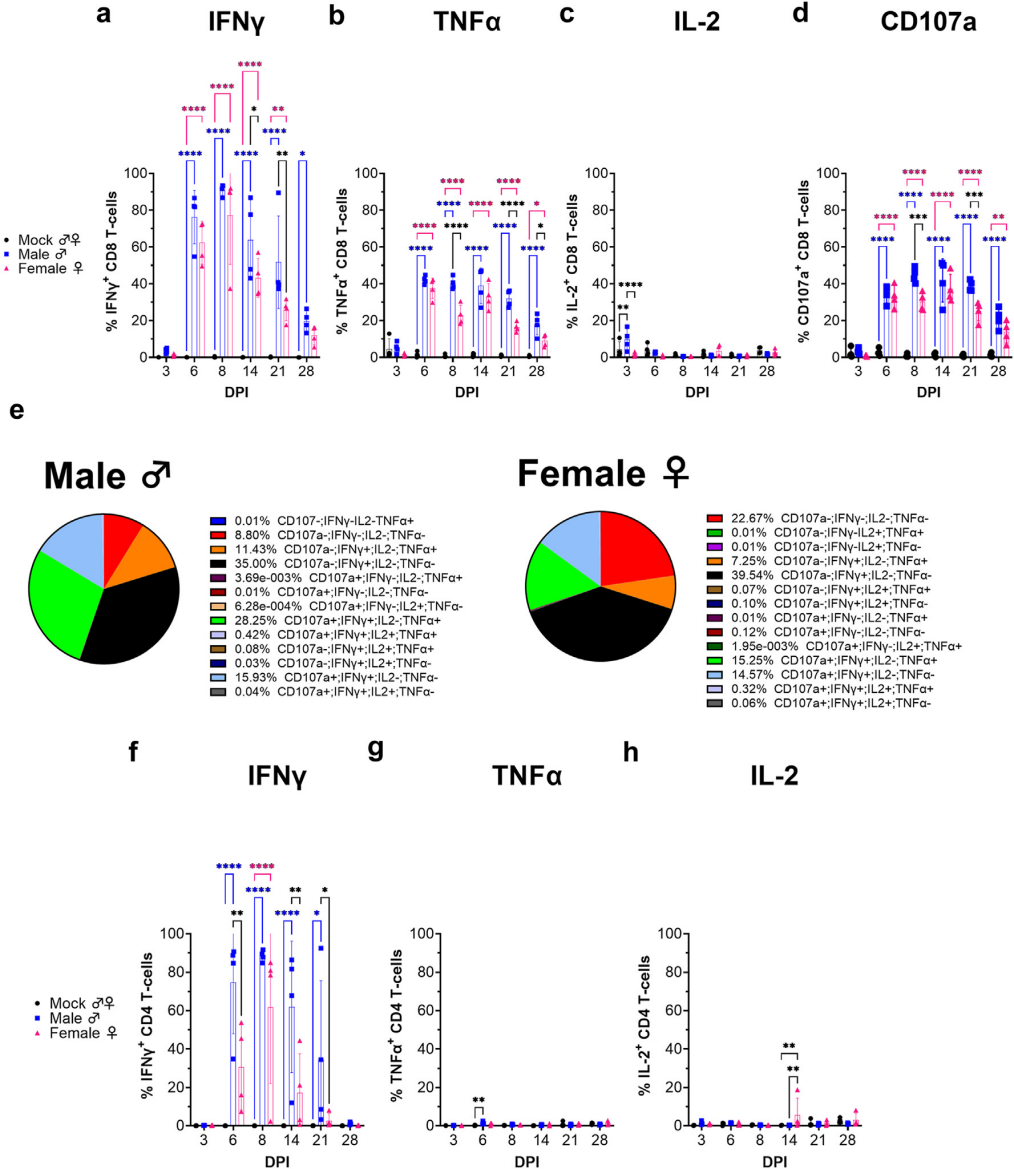
**CD8 T-cells in the livers of infected mice acquire CCHFV-specific effector functions.** Lymphocytes from the livers of mock or CCHFV-infected male or female mice were collected at indicated time points and stimulated with GPC peptide pool 10 *ex vivo* in the presence of antibody against CD107a and cytokine production after 6 h of stimulation measured by ICS. Data presented as percentage of parent population CD8^+^ T-cells (a–e) or CD4^+^ T-cells (f–h) positive for indicated cytokine or cytokines or CD107a. N = 4 mice per group. Data shown as mean plus standard deviation. Statistics calculated using a two-way ANOVA with Tukey's multiple comparisons test. ∗P < 0.05, ∗∗P < 0.01, ∗∗∗P < 0.001, ∗∗∗∗P < 0.0001.

Our data showed that pool 10 stimulated CD8^+^ T-cells in the spleen to produce IFNγ and we wanted to further characterize the CCHFV-specific response in the liver. We stimulated cells with pool 10 and performed ICS on lymphocytes collected from the liver at days 3, 6, 8, 14, 21 and 28 PI. The gating strategy is shown in Supplemental Figure S1. Consistent with our ELISpot data showing robust CCHFV-specific IFNγ response by day 6 in the spleen, by day 6 in the liver both male and female mice had significant increases in CCHFV-specific IFNγ^+^ CD8^+^ T-cells (Fig. 5a). By day 6, 76% of CD8^+^ T-cells in the livers of infected male mice produced IFNγ in response to CCHFV peptides and this increased to over 90% by day 8 PI (Fig. 5a). Over 75% of CD8^+^ T-cells from the livers of female mice produced IFNγ in response to CCHFV peptides by day 6 PI (Fig. 5a). Significant CCHFV-specific IFNγ production was measured until at least day 28 in male mice and day 21 in female mice (Fig. 5a). We also evaluated TNFα production in response to CCHFV peptides. Similar to IFNγ, robust increases in CCHFV-specific TNFα^+^ CD8^+^ T-cells was measured by day 6 PI in both male and female infected mice (Fig. 5b). In both male and female infected mice, significant increases in CCHFV-specific TNFα^+^ CD8^+^ T-cells was measured until at least day 28 (Fig. 5b). We also measured IL-2^+^ CD8^+^ T-cells. However, besides a slight but significant increase in IL-2^+^ CD8^+^ T-cells at day 3 in the livers of infected male mice, no differences were seen compared to mock infected mice (Fig. 5c). Lastly, our flow cytometry data indicated that most CD8^+^ T-cells expressed perforin by day 6 PI (Fig. 3g). To evaluate whether these CD8^+^ T-cells had CCHFV-specific cytotoxic activity, we performed a CD107a degranulation assay.^15^ In response to CCHFV peptides, by day 6 PI, over 30% of CD8^+^ T-cells from the livers of both male and female mice were CD107a^+^ (Fig. 5d) suggesting CD8^+^ T-cells rapidly acquired CCHFV-specific cytotoxic function. This response was maintained until at least day 28 in both male and female mice, consistent with sustained perforin expression in these mice.

We also evaluated the ability of CCHFV-specific CD8^+^ T-cells to produce multiple cytokines as T-cells that can produce multiple cytokines are thought to provide functionally superior antiviral control.^16^^,^^17^ At day 8 PI, in both male and female mice, the largest population of CD8^+^ T-cells, 35 and 39% respectively, produced only IFNγ in response to CCHFV peptides (Fig. 5e). However, in male mice, the majority of CD8 T-cells were polyfunctional with 28% expressing CD107a, IFNγ, and TNFα, 15% expressing CD107a and IFNγ, and 11% expressing IFNγ and TNFα (Fig. 5e). We measured similar populations in female mice, 15%, 14% and 7% respectively (Fig. 5e). A low percentage of T-cells in both male and female mice, 0.42% and 0.32% respectively, expressed CD107a, IFNγ, TNFα and IL-2 (Fig. 5e). These data indicate that a plurality of CCHFV-specific T-cells in both male and female mice rapidly acquire polyfunctional responses to CCHFV.

Our ICS data on splenocytes at day 8 showed that neither pool 9 nor 10 resulted in stimulation of splenic CD4^+^ T-cells. However, in contrast, liver CD4^+^ T-cells acquired CCHFV-specific activity. We measured significant increases in IFNγ^+^ CD4^+^ T-cells by day 6 PI in both male and female infected mice (Fig. 5f). We measured a slight but significant increase in TNFα^+^ CD4 T-cells in the livers of infected male mice at day 6 PI (Fig. 5g) but otherwise no significant differences were measured in percentages of TNFα^+^ or IL-2^+^ CD4^+^ T-cells in male or female mice (Fig. 5g and h). Our contrasting data between splenic and liver CD4^+^ T-cells suggest there exist tissue-specific activation states of CD4^+^ T-cells in CCHFV-infected mice.

In the absence of stimulation with CCHFV peptides, compared to mock-infected mice, we measured slight (<5%) but significant increases in IFNγ^+^ CD8^+^ T-cells at day 3, 6 and 8 PI and IFNγ^+^ CD4^+^ T-cells at days 6 and 8 in infected male mice (Supplemental Figure S3). We also measured a slight (<5%) but significant increase in IFNγ^+^ CD8^+^ T-cells in female mice at day 3 PI and IFNγ^+^ CD4^+^ T-cells at day 14 PI. This is likely due to activity against CCHFV present in the liver tissue at these timepoints (Fig. 2). No significant increases in TNFα^+^, IL-2^+^ or CD107a^+^ CD8^+^ T-cells were measured in unstimulated cells from CCHFV-infected mice at any timepoint (Supplemental Figure S3). Similarly, besides a slight but significant increase in IL-2^+^ CD4^+^ T-cells in infected female mice at day 14 PI, no other significant differences were measured in TNFα^+^ or IL-2^+^ CD4^+^ T-cells in male or female mice (Supplemental Figure S3). These data confirm that responses measured in cells stimulated with CCHFV peptides were responding in a CCHFV-specific manner.

Comparing infected male and female mice, in male mice we observed significantly greater percentages of IFNγ^+^ CD8^+^ and CD4^+^ T-cells at days 14 and 21 PI, greater percentages of TNFα^+^ CD8^+^ T-cells at days 8, 21 and 28 PI, and greater percentages of CD107a^+^ CD8^+^ T-cells at days 8 and 21 PI (Fig. 5a–d) suggesting male mice developed stronger cellular immunity against CCHFV. Cumulatively, our ICS data indicate that CD4^+^ and CD8^+^ T-cells rapidly acquire CCHFV-specific antiviral effector functions and worse clinical disease in male mice is not associated with diminished CCHFV-specific cellular immunity.

### Identification of the minimal CD8^+^ T-cell epitopes

**Fig. 6:**
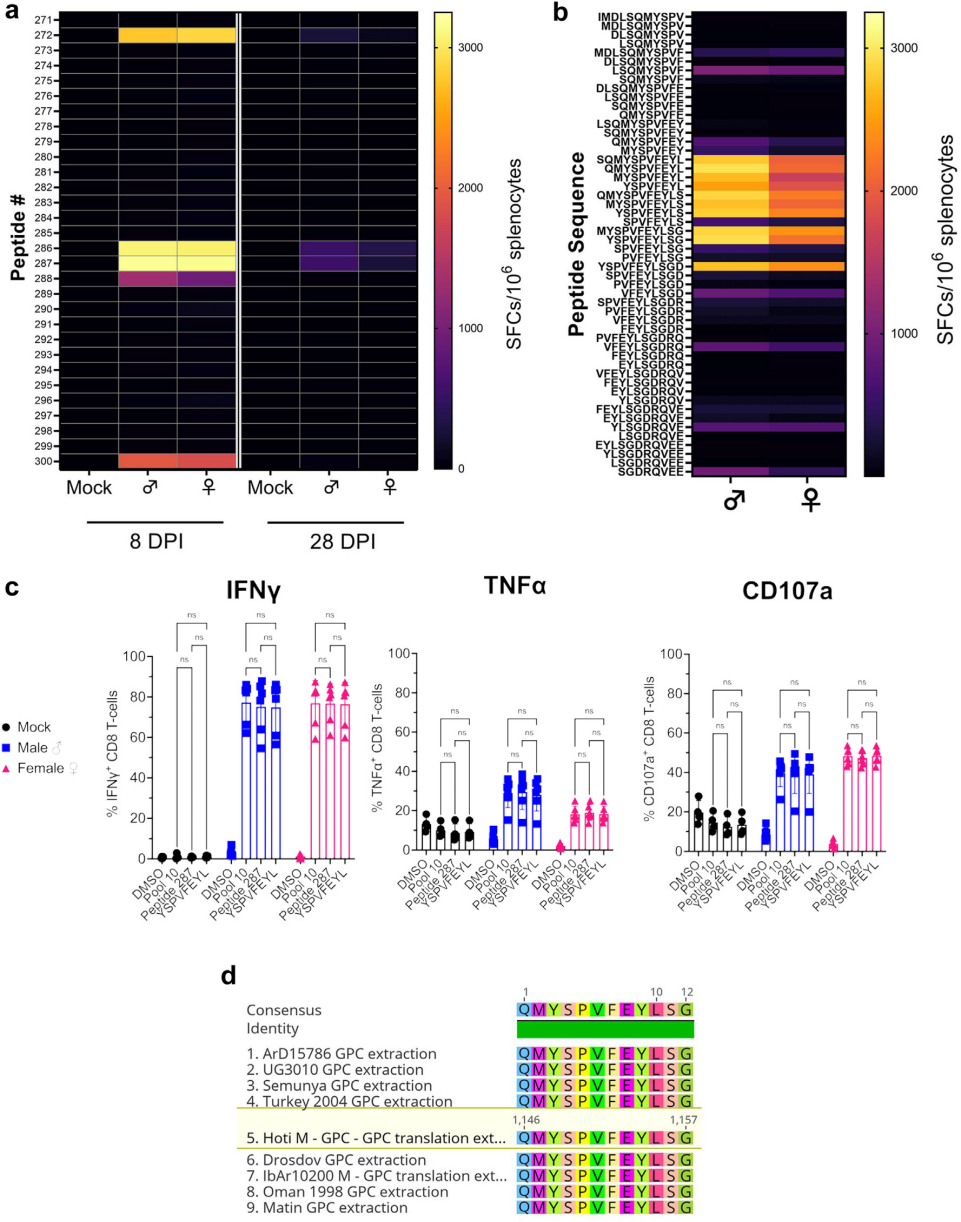
**Identification of the immunodominant CD8 epitope.** (a) Pooled splenocytes from four mock- or CCHFV-infected WT mice were collected at indicated DPI and CCHFV-specific T-cell responses against indicated peptides from the GPC pool 10 measured by IFNγ ELISpot. (b) Splenocytes from day 8 PI from CCHFV-infected male or female mice were stimulated with purified peptides of the indicated sequence and responses measured by IFNγ ELISpot. (a & b) Data presented as SFCs normalized to 10^6^ splenocytes and the average (mean) of duplicate measurements for each group and peptide. (c) Lymphocytes from livers of mock or infected male or female mice were collected at day 6 PI and stimulated with the indicated peptides *ex vivo* and IFNγ and TNFα production and degranulation (CD107a) measured by ICS. N = 4–6 per group. Statistics calculated using a two-way ANOVA with Tukey's multiple comparisons test. ns = P > 0.05. (d) Alignment of the identified minimal epitope across divergent CCHFV strains.

Our data showed that CD8^+^ T-cells were the principal effector cell population of cellular immunity against CCHFV and developed rapid and sustained CCHFV specific-effector functions. We therefore wanted to identify the minimal CD8 epitopes within GPC pool 10. We evaluated IFNγ ELISpot responses against the individual peptides comprising this pool by splenocytes collected at day 8 and 28 PI. IFNγ responses against pool 10 were directed mostly against the individual peptides 286 (GPC aa# 1141-IMDLSQMYSPVFEYL) and 287 (1145-SQMYSPVFEYLSGDR) with responses also against peptide 272 (1085-VSTANIALSWSSVEH) (Fig. 6a). Further, comparison of day 8 and 28 showed that while cellular immunity overall declined through day 28, the epitopes targeted remained consistent (Fig. 6a).

To identify the minimal epitopes, we performed IFNγ ELISpots on day 8 splenocytes using T-cell truncation libraries consisting of purified (>90%) 11- to 8-mers spanning the identified dominant peptide sequences in the Gc (peptides 286, 287 and 288) (Fig. 6b). Robust responses were measured against 11-mer peptides aa# 1145-SQMYSPVFEYL, 1146-QMYSPVFEYLS, 1147- MYSPVFEYLSG and 1148-YSPVFEYLSGD (Fig. 6b). Further mapping of this epitope through continued truncation identified the minimal epitope as an 8-mer 1148-YSPVFEYL that elicited robust IFNγ responses (2500 SFCs/10^6^ splenocytes in male mice) alone (Fig. 6b). A further increase in responses (>2800 SFCs/10^6^ splenocytes in male mice) was measured against longer peptides containing this core sequence (9-mers 1147-MYSPVFEYL, 1148-YSPVFEYLS, 10-mers 1146-QMYSPVFEYL, 1148-YSPVFEYLSG and the 11-mers mentioned above) (Fig. 6b) suggesting that the optimal Gc epitope is a 9 to 11-mer. This size is consistent with the size restriction and preference of MHC-I restricted epitopes^18^ and MHC-I binding predictions using the IEDB analysis resource (http://tools.immuneepitope.org/mhci/) that predicted YSPVFEYL would bind strongly to H-2Kb and with less affinity to H-2Db. We confirmed that this 8-mer sequence could stimulate CCHFV-specific CD8^+^ T-cells in the liver through ICS. Day 6 PI lymphocytes from the livers of mock or infected male and female mice were stimulated *ex vivo* with the YSPVFEVL peptide or peptide 287 containing this core sequence (Fig. 6c). Consistent with our ELISpot data, our ICS data showed that the core epitope 1148-YSPVFEYL or peptide 287 containing this core epitope could stimulate CD8^+^ T-cells as efficiently as pool 10 to produce IFNγ, TNFα and degranulate (CD107a^+^) in a CCHFV-specific manner (Fig. 6c).

Comparison of the epitope across diverse CCHFV strains from multiple clades showed that the core Gc epitope 1148-YSPVFEYL and the extended epitope 1146-QMYSPVFEYLSG are completely conserved across CCHFV strains ArD 15786, UG3010, Semunya, Turkey 2004, Drosdov, IbAr10200, Oman 1998, Matin and the parental strain of MA-CCHFV, strain Hoti (Fig. 6e). This would suggest that vaccination or infection of mice on the C57BL6/J background with diverse strains of CCHFV would result in similar targeting of YSPVFEYL.

### Mice deficient in IFNγ and perforin but not TNFα exhibit worsened disease

**Fig. 7:**
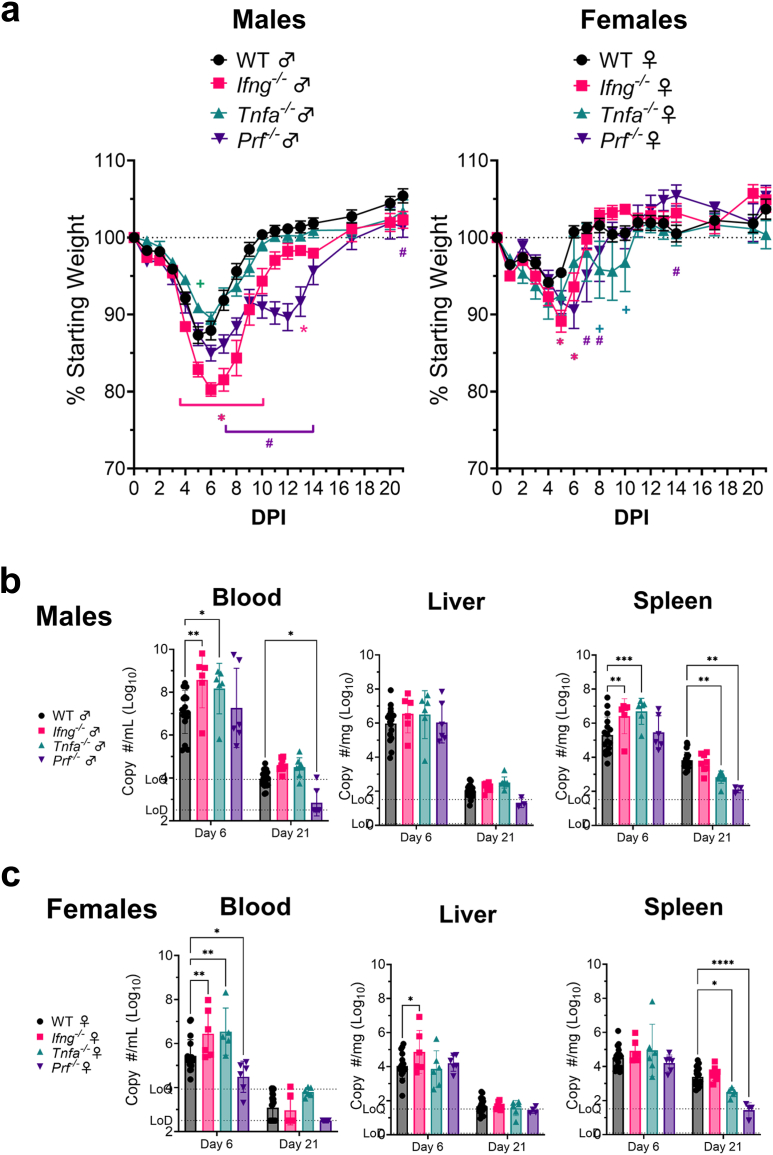
**Mice deficient in IFNγ and perforin but not TNFα**^**−/−**^**have worsened disease.** Male or female WT, Ifng^−/−^, *Tnfa*^−/−^ or *prf*^*−/−*^ mice were infected with MA-CCHFV. (a) Male and female mice were weighed daily. N = 24 for WT and N = 8 for *Ifng*^*−/−*^, *Tnfa*^*−/−*^ or *prf*^*−/−*^ for each sex. (b) Viral loads in indicated tissues from *Ifng*^*−/−*^*, Tnfa*^*−/−*^ or *prf*^*−/−*^ male (b) or female (c) mice were quantified by qRT-PCR. N = 16–20 for male or female WT mice. N = 3–8 for *Ifng*^*−/−*^*, Tnfa*^*−/−*^ or *prf*^*−/−*^. Data shown as mean plus standard error. Statistics were calculated with a two-way ANOVA with Dunnett's multiple comparisons test. ∗P < 0.05, ∗∗P < 0.01, ∗∗∗P < 0.001, ∗∗∗∗P < 0.0001.

Our data demonstrate that CD8^+^ T-cells in the livers of CCHFV-infected mice produce IFNγ, TNFα and perforin. We therefore investigated whether these effector functions were required to control the infection in MA-CCHFV infected WT mice. We infected male and female WT, *Ifng*^*−/−*^, *Tnfa*^*−/−*^ or *prf*^*−/−*^ mice with MA-CCHFV and evaluated clinical disease progression and viral loads in key tissues. Both male and female *Ifng*^*−/−*^mice exhibited worsened disease compared to WT mice characterized by increased and prolonged weight loss (Fig. 7a) although in all strains, female mice had milder disease than male mice. TNFα deficiency did not alter MA-CCHFV disease progression in male mice. Female *Tnfa*^*−/−*^ mice had slightly higher weight loss than WT females and displayed a biphasic disease pattern, with peak disease at day 5 (highest weight loss), recovery to day 8 when they again started to lose weight and then began recovery on day 10 post-infection. This biphasic disease pattern was also observed in male *prf*^*−/−*^ mice. Male *prf*^*−/−*^ mice had significantly worsened disease, indicated by higher and prolonged weight loss compared to their WT counterparts (Fig. 7a). The peak of the second phase of the disease was day 12, after which they began to recover from the infection (Fig. 7a). Female *prf*^*−/−*^ mice also had higher weight loss than WT mice, with peak clinical disease on day 6, although no biphasic disease was observed (Fig. 7a).

In agreement with the worsened disease in male and female *Ifng*^*−/−*^mice, significantly increased viral loads were measured in the blood and spleen of male, and blood and liver of female *Ifng*^*−/−*^ mice compared to WT mice (Fig. 7b and c). We also analyzed viral loads at day 8 PI, peak disease in *Ifng*^*−/−*^ mice and saw significantly increased viral loads in the liver and spleen in male mice (Supplemental Figure S3). Although disease progression in male and female *Tnfa*^*−/−*^ mice was similar to WT mice, significantly increased viral loads in the blood compared to WT mice were observed at day 6 post-infection (Fig. 7b and c). At day 21 PI, both male and female *Tnfa*^*−/−*^ mice had significantly lower viral loads in the spleen (Fig. 7b and c). Interestingly, worse disease observed in *prf*^*−/−*^ mice did not correlate with higher viral loads. On the contrary, female *prf*^*−/−*^ mice had significantly decreased viral loads in the blood compared to WT mice at day 6 PI, male *prf*^*−/−*^ had significantly reduced viral loads at day 21 PI and both male and female *prf*^*−/−*^ had significantly reduced viral loads in the spleen at day 21 PI. Together these data suggest that IFNγ and TNFα but not perforin are required for control of CCHFV during the acute disease, while TNFα and perforin may impair clearance of the infection at later timepoints.

## Discussion

In this report we investigated the role of cellular immunity in control of acute CCHFV infection, a key but understudied area for CCHFV pathogenesis. We identified an important role for CD8^+^ T-cells in efficient control of the infection. CD8^+^ T-cells were rapidly activated in response to the infection, increasing in number and acquiring CCHFV-specific antiviral effector functions. Our data suggest that the principal effector function of CD8^+^ T-cells in control of MA-CCHFV replication is production of antiviral cytokines such as IFNγ. By day 8 PI, >70% of CD8^+^ T-cells in both male and female mice produced IFNγ in response to CCHFV peptides. This magnitude of response is similar to what has been reported for LCMV in which 80% of CD8^+^ T-cells were specific for just 10 viral peptides.^19^^,^^20^ Further, this is consistent with our previous findings in *Ifnar*^*−/−*^ mice infected with a human clinical isolate of CCHFV.^10^^,^^21^ In that model, depletion of CD8^+^ T-cells or antibody blockade of IFNγ lead to worsened survival.^10^ However, in contrast to our findings here, in the *Ifnar*^*−/−*^ model depletion of CD4^+^ T-cells also worsened survival and the systemic IFNγ response was largely mediated by CD4^+^ T-cells.^10^ These differences may be due to lack of type I IFN signaling in our previous studies^22^ and survival in T-cell depleted mice likely requires humoral immunity as we have previously shown that MA-CCHFV infection of mice deficient in both B- and T-cells is rapidly lethal.^12^

We identified CCHFV-specific production of IFNγ and TNFα. Both IFNγ and TNFα are pleiotropic cytokines with multiple immunomodulatory effects. IFNγ has a multitude of antiviral effects including recruitment of immune cells, upregulating antigen presentation, direct inhibition of viral replication through induction of ISGs, promotion of apoptosis and induction of antimicrobial effector functions.^23^ Like IFNγ, TNFα plays a critical role in the control of viral infections including enhanced antigen presentation, recruitment of leukocytes, apoptosis of infected cells, a direct antiviral effect, and polarization of T_H_ cell responses.^24^ How IFNγ and TNFα contribute to control of CCHFV infection will require further study but our data suggest that IFNγ and TNFα both contribute to control of viral replication at early timepoints as increased viral loads were measured in mice deficient in these cytokines.

Notably, our findings are in contrast to a recent report in CCHFV-infected type I IFN deficient mice that suggested TNFα may be pathogenic.^25^ In that study by Golden et al., they found that infecting mice treated with an antibody to block type I IFN and lacking the TNFα receptors, or treatment with a TNFα neutralizing antibody in a post-exposure setting resulted in improved survival and lower weight loss. An explanation for the distinct results between our study and the study by Golden et al. is that our studies used an immunocompetent mouse model with intact type I IFN responses, avoiding potential confounding factors of type I IFN deficiency on TNFα responses.^26^^,^^27^ Further, elevated levels of IFNγ and TNFα are often reported in human CCHF cases and high levels may correlate with fatal outcome.^28^, ^29^, ^30^, ^31^, ^32^ MA-CCHFV infection of WT mice is only occasionally lethal and was not associated with systemically high levels of IFNγ or TNFα,^12^ while the model used by Golden et al. is nearly uniformly lethal and associated with increased levels of TNFα and IFNγ.^25^ Supporting this hypothesis, we recently reported a lethal model using a high-dose MA-CCHFV challenge (10^5^ TCID50 versus the 10^4^ TCID50 used here). In that model significantly increased levels of TNFα were measured in lethally infected mice.^33^ Additionally, Golden et al. found strain-specific differences in inflammatory cytokine induction, with strain Hoti, the parental strain of MA-CCHFV, inducing less inflammation than the Afg-09 strain.^25^ Thus, TNFα and other inflammatory cytokines may have distinct contributions to CCHF outcome dependent on disease severity, intact or absent type I IFN signaling and CCHFV-strain specific interactions with host inflammatory pathways.

Although we identified CCHFV-specific production of these cytokines by T-cells, both can be produced by a multitude of cell types including cells that are targets of CCHFV infection such as macrophages and Kupfer cells.^12^^,^^23^^,^^34^^,^^35^ In other viral hemorrhagic fevers, infection of these cell types can lead to dysregulated cytokine production.^36^^,^^37^ Thus, it is unclear if the systemic elevation of these cytokines in severe CCHF cases represents ongoing immunopathology or instead, otherwise protective immune responses attempting to control overwhelming viral replication. Nevertheless, in our studies, broad genetic deletion of IFNγ and TNFα did not improve disease demonstrating that severe CCHFV-mediated pathology can occur in the absence of these cytokines.

Apart from production of cytokines, we found that CD8^+^ T-cells rapidly upregulate perforin and degranulate in a CCHFV-specific manner. Interestingly, infection of mice deficient in perforin, a key molecule for cytotoxic T-cell function,^38^ resulted in a prolonged, biphasic disease that was not associated with impaired viral control in key tissues such as the liver and spleen. This suggests perforin deficiency results in a pathogenic host immune response to MA-CCHFV. The role of perforin in CCHF is unclear. In a study of CCHFV-infected humanized mice, elevated perforin levels were observed in CD8^+^ T-cells of terminal mice.^39^ A similar disease phenotype was also observed in the *Tnfa*^*−/−*^ female mice but was associated with impaired viral control at early timepoints but improved control at later timepoints. Intriguingly, both perforin and TNFα are involved in regulating the cellular immune response^40^, ^41^, ^42^ and have been linked to hemophagocytic lymphohistiocytosis (HLH), an inflammatory condition seen in humans.^43^^,^^44^ LCMV infected *Prf*^−/−^ mice develop splenomegaly, liver pathology, excessive cytokine production and eventual death.^45^^,^^46^ This condition in humans is associated with genetic defects in the perforin gene^43^ or can be due to immunological stimuli such as an otherwise innocuous viral infection.^47^ While the relationship between perforin deficiency and HLH has been established, there are paradoxical reports of the association of TNFα and HLH, with cases of successful treatment of refractory HLH with TNFα inhibitors^44^^,^^48^ as well as cases of development of HLH after treatment with TNFα inhibitors^49^^,^^50^ being reported. IFNγ may also contribute to HLH. In mouse models, CD8^+^ T-cells and IFNγ were required for development of HLH^45^ and an anti-IFNγ antibody was approved by the FDA to treat HLH resistant to conventional treatments.^51^ There is substantial overlap in the clinical presentation of HLH and viral hemorrhagic fevers, such as CCHF (including liver pathology, thrombocytopenia, inflammatory cytokines, hemorrhage, DIC)^52^^,^^53^ and HLH in CCHF cases has been reported^54^^,^^55^ leading to speculation that VHFs may induce HLH-like disease. MA-CCHFV may provide a model to investigate the contribution of HLH-like severe disease caused by VHFs.

Lastly, we identified the immunodominant CD8 epitope in MA-CCHFV infected mice, YSPVFEYL. This 8-mer peptide is located within the viral Gc protein. In vaccine studies using a replicating RNA vaccine, similar peptide pools were targeted^11^ and this YSPVFEYL epitope is highly conserved suggesting a similar epitope would be targeted by vaccination or infection of C57BL6/J mice with divergent strains of CCHFV. Further studies will be needed to determine if this narrow focus of cellular immunity on the CCHFV Gc is an artifact of the inbred C57BL6/J mice used here. Vaccination of non-human primates and other strains of mice resulted in different epitopes being targeted^56^, ^57^, ^58^ and human infections resulted primarily in responses against the NP.^9^ We also cannot exclude the possibility that mouse-adaptation of CCHFV resulted in a virus that elicits distinct T-cell immunity from clinical isolates. However, the mutations in MA-CCHFV were rapidly selected for during passage in *Rag1*^*−/−*^ mice which lack any adaptive immunity suggesting MA-CCHFV was not selected for its ability to evade or overcome cellular immunity. Nevertheless, identification of the dominant CD8^+^ T-cell epitope in MA-CCHFV-infected C57BL6/J mice will provide a powerful tool for continued study of CCHFV-specific T-cells in this and other mouse models of CCHF.

### Limitations of our study

Similar to what we reported in the initial description of the MA-CCHFV model,^12^ male mice exhibited significantly worse clinical disease than even immunodeficient female mice. However, we must highlight that these large differences in disease outcome between male and female mice are not reported in humans infected with CCHFV. Instead, the differences in male and female mice infected with MA-CCHFV provide an opportunity to compare and contrast host responses that are associated with mild disease and those associated with severe disease. This may shed light on the mechanisms that mediate the spectrum of disease observed in CCHFV-infected humans. Although sex differences in host immunity are well established^59^ and our data indicate cellular immunity is an important host response, our data in male mice also demonstrate that severe disease can occur despite robust cellular immune responses. This suggests that additional host responses contribute to disease outcome upon infection with CCHFV and we did not examine them here. Ongoing studies are investigating humoral and innate immunity to determine their contribution to disease outcome in MA-CCHFV infected mice. Further, we only examined T-cell responses to MA-CCHFV and other strains of CCHFV may have differing host and viral determinants of outcome. Lastly, our results and those of others in mouse models will need to be compared with findings in other models such as non-human primates and findings in human cases of CCHF.

Cumulatively our data indicate that cellular immunity principally through CD8^+^ T-cells and antiviral cytokine production is required for effective control of acute CCHFV infection. Rapid and robust CD8^+^ T-cell activation was measured in the liver and spleen and CD8^+^ T-cells rapidly acquired CCHFV-specific antiviral effector functions. Control of CCHFV is likely mediated through production of IFNγ or TNFα and other antiviral cytokines as mice deficient in perforin did not exhibit worsened viral control. We also identified the immunodominant CD8^+^ T-cell epitopes within the CCHFV GPC enabling further studies of CCHFV-specific T-cell functions. Our data add to our understanding of how host adaptive immunity contributes to control of CCHFV infection.

## Contributors

DR, KMW, SL, EM, AC, DWH performed experiments. DR and DWH data curation, formal analysis and data verification. DR, HF and DWH writing–original draft, review and editing. DWH and HF supervision. HF project administration, resources and funding acquisition. All authors have read and approved the final version of the manuscript.

## Data sharing statement

Data are available upon reasonable request.

## Declaration of interests

Authors have no conflicts of interest to report.

## Appendix A Supplementary data

Supplementary Table S1 Statistical comparisons of telemetry data in figure 1. Statistical comparison was performed by comparing indicated day post-infection to baseline values on day 0. Two-Way ANOVA with Dunnett’s multiple comparisons test. ns = P >0.05, ∗ P < 0.05, ∗∗ P< 0.01, ∗∗∗ P < 0.001, ∗∗∗∗ P < 0.0001.
