# Supplementary material for: CD8+ T-cells target the Crimean-Congo haemorrhagic fever virus Gc protein to control the infection in wild-type mice
Source: eBioMedicine. 2023 Oct 20;97:104839. doi: 10.1016/j.ebiom.2023.104839 (PMC10623175; doi:10.1016/j.ebiom.2023.104839)
Supplement: Supplementary Figures [file mmc2.pdf]

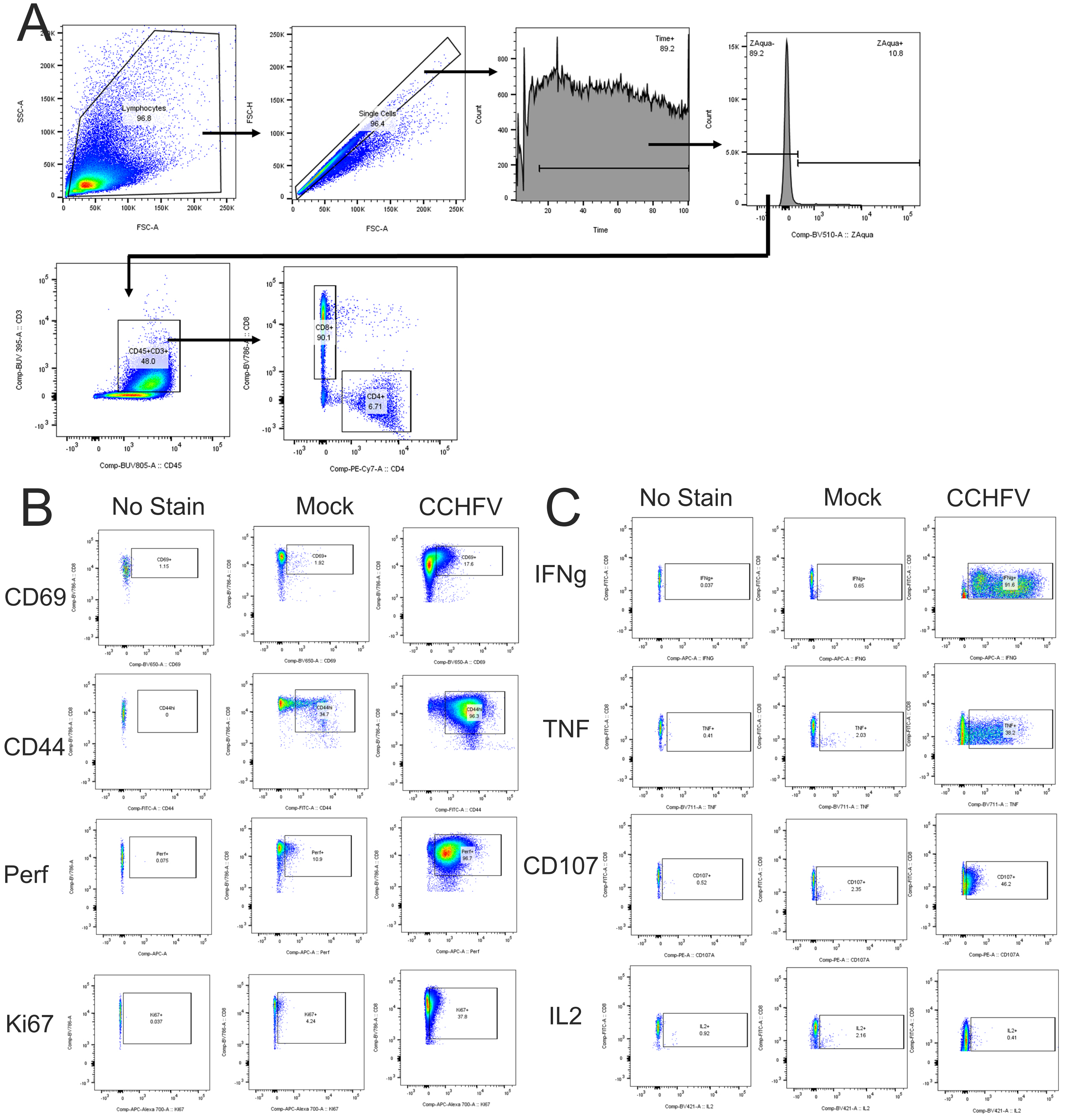

Supplemental Figure 1: Flow gating strategy. (A) Cells were gated on FSC and SSC to exclude debris and doublets. A time gate was used to exclude early events and dead cells were excluded by ZombieAqua viability dye. T cells were then gated on CD45+CD3+. CD4+ or CD8+ T-cells were then analzyed for activation markers (B) or cytokine production after stimulation ex vivo (C).

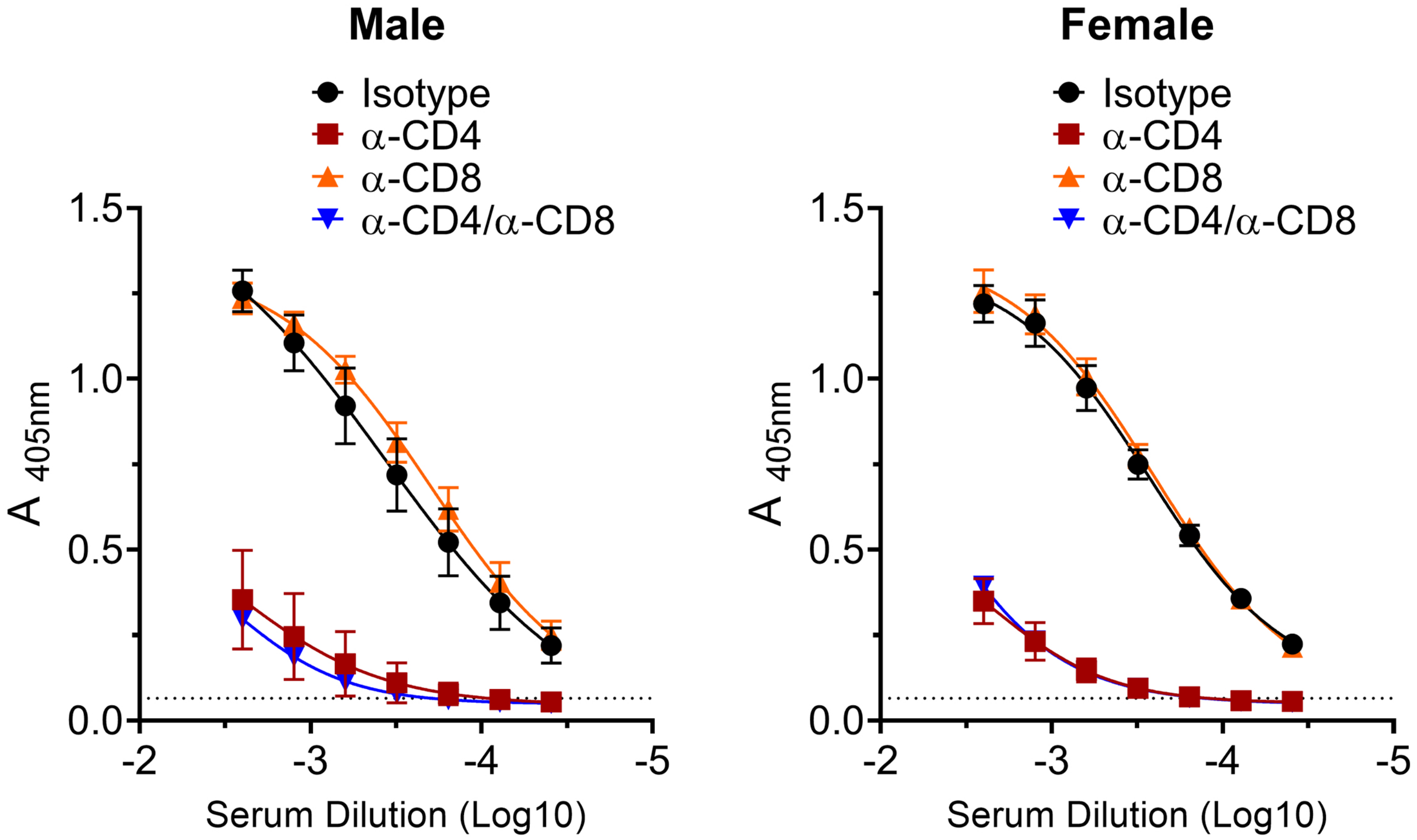

Supplemental Figure 2: Humoral immunity to CCHFV is CD4 T-cell dependent. MA-CCHFV infected male or female mice were treated with antibodies to deplete the indicated cell types or isotype control. At day 21, serum was collected and a whole-virion ELISA performed to quantify CCHFV-specific IgG.

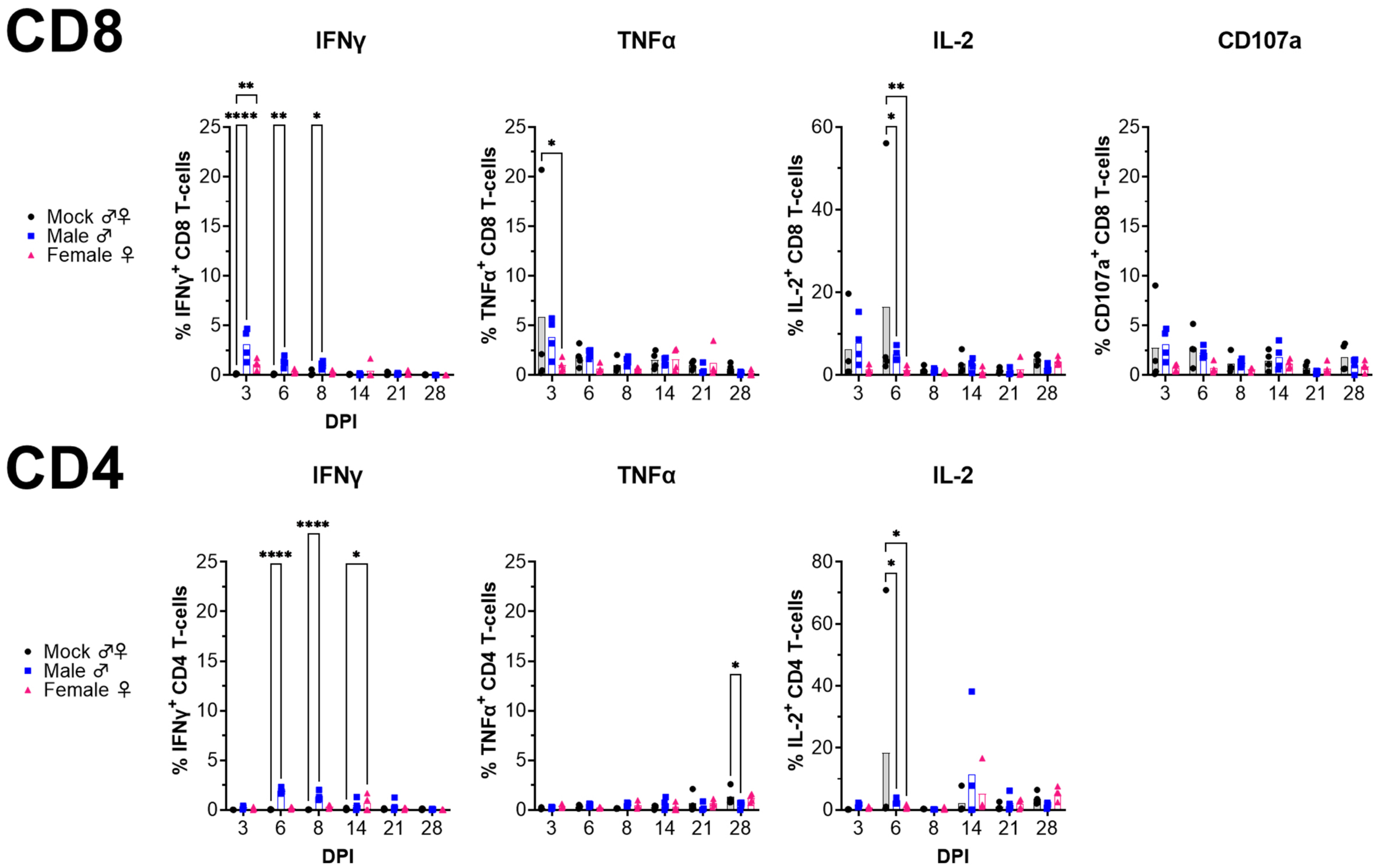

Supplemental Figure 3: Minimal cytokine production in absence of CCHFV peptide stimulation. Lymphocytes from the livers of mock or CCHFV-infected male or female mice were collected at indicated time points and stimulated with DMSOex vivo in the presence of antibody against CD107a and cytokine production after six hours of stimulation measured by ICS. Data presented as percentage of parent population CD8 T-cells (A – D) or CD4 T-cells (E – G) positive for indicated cytokine or CD107a. Data shown as mean plus standard deviation. Statistics calculated using a two-way ANOVA with Tukey’s multiple comparisons test. * P < 0.05, ** P < 0.01, *** P < 0.001, **** P < 0.0001.
